# Feasibility Study of a Wearable Exoskeleton for Children: Is the Gait Altered by Adding Masses on Lower Limbs?

**DOI:** 10.1371/journal.pone.0073139

**Published:** 2013-09-04

**Authors:** Stefano Rossi, Alessandra Colazza, Maurizio Petrarca, Enrico Castelli, Paolo Cappa, Hermano Igo Krebs

**Affiliations:** 1 DEIM Department of Economics and Management – Industrial Engineering, University of Tuscia, Viterbo, Italy; 2 MARLab Movement Analysis and Robotics Laboratory – Neuroscience and Neurorehabilitation Department, “Bambino Gesù” Children’s Hospital, Rome, Italy; 3 Department of Mechanical and Aerospace Engineering, “Sapienza” University of Rome, Rome, Italy; 4 Department of Mechanical Engineering, Massachusetts Institute of Technology, Cambridge, Massachusetts, United States of America; 5 Department of Neurology and Division of Rehabilitative Medicine, University of Maryland School of Medicine, Baltimore, Maryland, United States of America; University of California, Merced, United States of America

## Abstract

We are designing a pediatric exoskeletal ankle robot (pediatric Anklebot) to promote gait habilitation in children with Cerebral Palsy (CP). Few studies have evaluated how much or whether the unilateral loading of a wearable exoskeleton may have the unwanted effect of altering significantly the gait. The purpose of this study was to evaluate whether adding masses up to 2.5 kg, the estimated overall added mass of the mentioned device, at the knee level alters the gait kinematics. Ten healthy children and eight children with CP, with light or mild gait impairment, walked wearing a knee brace with several masses. Gait parameters and lower-limb joint kinematics were analyzed with an optoelectronic system under six conditions: without brace (natural gait) and with masses placed at the knee level (0.5, 1.0, 1.5, 2.0, 2.5 kg). T-tests and repeated measures ANOVA tests were conducted in order to find noteworthy differences among the trial conditions and between loaded and unloaded legs. No statistically significant differences in gait parameters for both healthy children and children with CP were observed in the five “with added mass” conditions. We found significant differences among “natural gait” and “with added masses” conditions in knee flexion and hip extension angles for healthy children and in knee flexion angle for children with CP. This result can be interpreted as an effect of the mechanical constraint induced by the knee brace rather than the effect associated with load increase. The study demonstrates that the mechanical constraint induced by the brace has a measurable effect on the gait of healthy children and children with CP and that the added mass up to 2.5 kg does not alter the lower limb kinematics. This suggests that wearable devices weighing 25 N or less will not noticeably modify the gait patterns of the population examined here.

## Introduction

Cerebral Palsy (CP) affects at least 2 in 1,000 children born in Western countries [1] and this number might increase as perinatal deaths and intrapartum injuries have been decreasing, leading to growth in the survival rate of premature babies [2], [3]. CP significantly impacts motor performance, leading to deficits in muscle force generation and increases in muscle stiffness so that gait is marked by slow speed and disturbed motor control [4].

The proportion of non-walking children with CP has been stable over the last 20 years and across countries, despite the changes that have occurred in neonatal care throughout Europe [5]. Systematic study reviews on the effectiveness of physical therapy interventions in children with CP show limited evidence of effectiveness with few randomized controlled clinical trials [6]. Nevertheless, it has been demonstrated that high-intensity and task-specific programs have resulted in improved strength and functional performance that were sustained over time [7], [8].

During the last five decades, researchers have been developing lower extremity orthosis that are either passive or active to help impaired individuals to seek the most optimal gait given their pathology and to maximize their stability and safety. Detailed descriptions of developed exoskeletons are reported elsewhere, where a general framework for the study, classification and control algorithms of these devices can be found [9]–[11]. Robotic exoskeletons worn during gait in adults with paraplegia have the potential to be used in children and adults with CP [12]–[18]. Robotic therapy delivers a highly reproducible and high intensity training experience, affording the potential to integrate concepts of motor learning while quantitatively monitoring and adapting demands to the child’s progress. Moreover, many works pointed out the efficacy of robotic therapy in patients with CP [19]–[22].

Nowadays, the available computational power and the level of electronics miniaturization do not pose important problems in controlling wearable exoskeletons. One of the most difficult problems that still requires attention is the development of lightweight devices to avoid encumbering the movement ability of the neurologically impaired persons. Thus, before the design of a wearable exoskeleton, the study of the maximum device mass that the patients can support during the gait emerges. Few studies have addressed the impact of unilateral loading on the legs of healthy adult subjects [23], [24] and adult patients [25], [26]. Barnett et al. (1993) studied the effects of ankle weight addition (0.91, 1.82, and 2.73 kg) on gait and they found a linear correlation between decrease in walking speed and increase in the added mass [23]. Noble et al. (2006) analyzed healthy subjects walking on a treadmill with a 2 kg mass placed over the bulk of the muscle mass of the calf of their non-dominant leg. They concluded that it increased the loaded leg hip flexion angle and decreased the knee flexion angle during the swing phase [24]. Regnaux et al. (2008) attached a 2 and 4 kg mass, for a female and male respectively, around the less affected ankle of stroke patients walking on a treadmill and observed increases in the knee and hip excursions and improvement in motor performances including walking speed, step length, and cadence after the treadmill session [25]. Khanna et al. (2010) found that the gait pattern of stroke patients was not significantly altered if an unpowered robotic device of 3.6 kg was mounted anteriorly and proximally to the paretic leg during overground and treadmill gait. The presence of the robot load reduced the knee peak flexion and the ankle peak dorsiflexion but the spatiotemporal parameters were not altered [26]. The above mentioned studies examined changes in the gait pattern of adults and there are no equivalent studies on either normally developed children or children with CP.

We are presently developing a pediatric version of the adult Anklebot [15] and the purpose of the present study is to determine if a knee brace and additive masses up to 2.5 kg affect the gait pattern of normally developed children and children with CP. Ultimately our goal is to determine the target specification for the above mentioned pediatric robotic devices. Specifically, we analyzed the kinematics of lower limbs loaded unilaterally with five different masses (0.5, 1.0, 1.5, 2.0, and 2.5 kg) placed on the proximal third of the leg during gait. The results reported herewith will also be useful to other research groups that will be involved in the design of wearable lower limb exoskeletons for children.

## Methods

### Ethics Statement

Informed consent, in written form, was obtained from the parents of all children who were involved in the study. The protocol and the consent procedure were approved by the Ethics and Medical Board of the “Bambino Gesù” Children’s Hospital. The protocol conforms to the ethical standards laid down in the 1964 Declaration of Helsinki.

### Subjects

Eight children aged 5–9 years with CP (4 females and 4 males), body mass range of 20–29 kg (mean 25 kg) and height range of 1.04–1.38 m (mean 1.21 m) were enrolled in this study at the “Bambino Gesù” Children’s Hospital. The inclusion criteria were: mild spastic hemiplegia with levels I and II of Gross Motor Functional Classification System (GMFCS) [27]; no evident reduction in cognitive functions; ability to walk without assistive devices; comprehension of the verbal commands; absence of visual impairment; and no neurological or orthopedic surgery in the patient’s history.

Ten age-matched normally developed children (5 females and 5 males) with body mass range of 19–30 kg (mean 24 kg) and height range of 1.07–1.39 m (mean 1.24 m) were enrolled. The inclusion criteria were: no neurological or orthopedic impairments; no history of learning disabilities; and absence of visual impairment.

### Procedure

Testing was performed at the MARLab - Movement Analysis and Robotic Laboratory of the “Bambino Gesù” Children’s Hospital. The gait analyses were carried out in a large room with a 10 m gait path.

**Figure 1:**
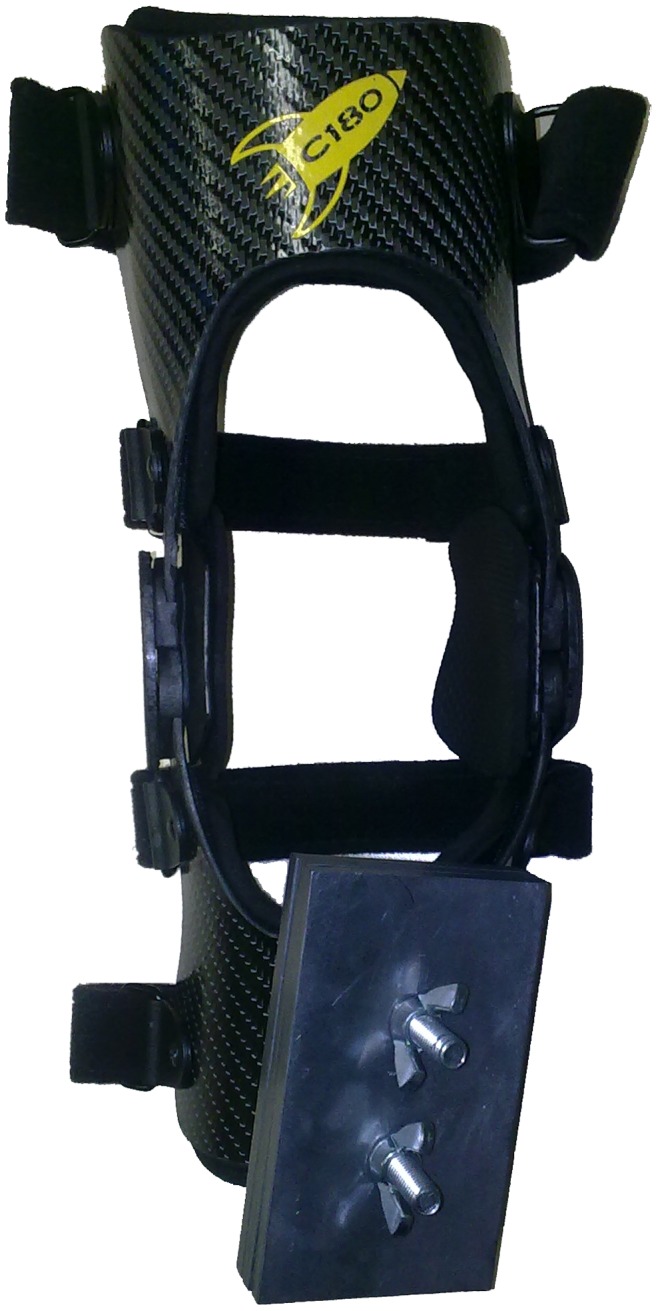
Knee brace. Knee brace and lead masses.

Subjects were asked to wear a commercial knee brace (C180 Sports Rocket – Ossur, USA) where several lead masses could be placed on the anterior and proximal third of the leg (Figure 1). The mass of the knee brace and each lead weight was 0.5 kg. The knee brace dimensions were: height equal to 29 cm and maximum width of 10 cm. We set the mass position according to the design of the adult version of the Anklebot [15], that is, mounted on a knee brace with the weight concentrated on the leg. The brace was positioned on the more affected side of the children with CP and on the non-dominant side of healthy subjects. The resulting outcome in the group with CP was assessed by clinical evaluation conducted by an expert physical therapist. The second group’s non-dominant side assessment was done by asking healthy subjects to kick a ball.

Six different unilateral loading conditions were examined. First, subjects walked without a knee brace and the “natural” unencumbered gait was evaluated (natural gait, NG condition). The second condition consisted of walking wearing the knee brace (0.5 kg). We added, in sequence, four lead masses onto the knee brace for an overall unilateral loading of 1.0 to 2.5 kg. We selected 2.5 kg as highest limit because it represented the 10% of the average body mass of subjects. Moreover, from a preliminary design of Anklebot pediatric version based on a scaling of adult version one, we estimated 2.5 kg as the total mass of the device by means of a 3D-CAD design engineering software [28].

Subjects were instructed to walk barefoot at a comfortable and self-selected speed. Subjects walked for at least 30 meters before each session to allow them to adapt to added masses; this distance was selected during “dry-tests” and deemed adequate for all subjects. Subjects rested in seated position between tasks for 5 minutes as the lead weights were being changed; all normally developed subjects and patients with CP completed the tasks without expressing fatigue. For each trial condition, data were collected during 5 walking bouts.

### Data Acquisition and Analysis

Kinematic data were recorded using an 8-camera VICON system (MX camera-workstation, Nexus 1.7 software, 200 Hz, PlugInGait marker set based on the Davis’ protocol [29]). More precisely, sixteen retro-reflective markers were placed on the subject’s skin surface as follows: posterior and anterior iliac spines (4 markers), lateral epicondyles (2 markers), thighs (2 markers), lateral malleoli (2 markers), legs (2 markers), second metatarsal head (2 markers), and calcaneous (2 markers). During “with added masses” conditions, markers placed on the lateral epicondyles were positioned, instead, on the knee brace joints in order to make them visible to the cameras. It is worthy to note that the reattachment procedure did not affect the estimation of gait kinematics taking care to conducting a new subject calibration before the “with added masses” gait sessions. In fact, in PluginGait protocol [29], the epicondyle marker is used to define the flexion-extension axis of knee and the center of knee joint (KJC) by means of the evaluation of “knee offset” as the semi-sum of knee width and marker diameter. In particular, after the reattachment procedure, the definition of knee rotation axis was guaranteed taking care to place the epicondyle marker on the flexion-extension axis of knee brace and the position of KJC was not altered evaluating the “knee offset” as the semi-sum of knee brace width and marker diameter. The marker trajectories were filtered with a Woltring filter - size 30 [30], [31]. Static and dynamic calibration tests, performed in accordance with the manufacturer’s indications, were conducted before each participant’s trial session and they showed that overall RMS error of marker coordinates in three-dimensional space was less than 1 mm. Trials were also videotaped in frontal and lateral planes.

The collected data were organized into two subsets: spatiotemporal parameters and kinematic data.

The spatiotemporal parameters were: stance phase (SP), single (SP_s) and double (SP_d) support phase, step length (SL), walking speed (WS), and symmetry index (SI) defined as [32]: 
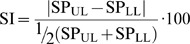
(1)where SP_UL_ and SP_LL_ are the stance phases of the unloaded (UL) and loaded (LL) leg, respectively; in NG trial, LL represents both the non-dominant leg for healthy subjects and the more affected limb of children with CP. The SI value represents the magnitude of asymmetry between legs during the gait. The SI value ranges from 0 to 200% and higher values represent a greater difference between the two sides.

Kinematic data included hip, knee, and ankle angles on the sagittal plane: the peak value of hip flexion (HF) and extension (HE) angles; peak (KF_max_) and lowest (KF_min_) values of knee flexion angle; and, finally, peak value of ankle dorsiflexion (AD) and plantarflexion (AP) angles.

### Statistical Analysis

All data were tested for normality with the Shapiro-Wilk test. One-way Repeated Measures ANOVA tests were conducted in order to find noteworthy differences among the six trial conditions both for loaded leg (LL) and unloaded leg (UL). Statistical significance was set at 0.05. When significance was found, a paired t-test procedure with a Bonferroni correction was performed in order to detect significant differences among trial conditions. Two-tailed t-tests were conducted to find any significant difference between LL and UL for each trial condition. No statistical comparison was conducted in order to find differences between healthy and CP subjects.

## Results

### Spatiotemporal Parameters

Table 1 and Table 2 show the SP, SP_s, SP_d, SL, WS and SI as a function of the added mass. For normally developed children, no significant differences were found among the six trial conditions and between LL and UL for each spatiotemporal parameter. For the subjects with CP, only the difference (p<0.01) between LL and UL for SP and SP_s was found. The SI data were always greater for CP than healthy subjects.

**Table 1:** Spatiotemporal parameters of loaded and unloaded leg for healthy children.

Healthy children	Leg	Trial conditions											
		NG		0.5 kg		1.0 kg		1.5 kg		2.0 kg		2.5 kg	
		*Mean*	*SD*	*Mean*	*SD*	*Mean*	*SD*	*Mean*	*SD*	*Mean*	*SD*	*Mean*	*SD*
SL [m]	LL	0.54	0.05	0.56	0.06	0.56	0.05	0.55	0.05	0.53	0.05	0.55	0.04
	UL	0.55	0.06	0.57	0.05	0.57	0.04	0.54	0.04	0.56	0.05	0.56	0.03
SP [%]	LL	57.7	2.4	57.8	2.8	56.9	2.3	57.6	2.6	56.6	2.8	55.9	2.9
	UL	58.0	2.2	58.0	2.7	58.5	2.1	58.6	1.5	58.8	1.4	58.7	1.1
SP_s [%]	LL	41.9	2.3	41.9	2.7	41.5	1.8	41.3	1.8	41.2	1.5	41.2	2.2
	UL	42.8	2.3	42.2	2.9	43.4	2.7	42.1	2.6	43.8	3.1	43.8	3.1
SP_d [%]		15.4	4.0	15.9	4.8	15.3	4.0	16.4	3.9	15.5	3.7	14.8	3.9
SI [%]		3.0	2.4	3.6	3.1	3.3	3.0	3.5	2.0	5.1	3.5	5.5	3.5
WS [m/s]		1.23	0.18	1.25	0.22	1.27	0.20	1.20	0.14	1.14	0.11	1.13	0.15

Mean and standard deviation for the spatiotemporal parameters of loaded (LL) and unloaded (UL) leg for healthy children. During gait without added masses (NG), LL represents the non-dominant limb of healthy subjects. No statistical differences were found.

**Table 2:** Spatiotemporal parameters of loaded and unloaded leg for children with CP.

Children with CP	Leg	Trial conditions											
		NG		0.5 kg		1.0 kg		1.5 kg		2.0 kg		2.5 kg	
		*Mean*	*SD*	*Mean*	*SD*	*Mean*	*SD*	*Mean*	*SD*	*Mean*	*SD*	*Mean*	*SD*
SL [m]	LL	0.45	0.08	0.42	0.10	0.49	0.07	0.45	0.08	0.42	0.08	0.43	0.06
	UL	0.44	0.06	0.45	0.07	0.47	0.08	0.43	0.07	0.45	0.06	0.46	0.04
SP [%]	LL	**56.9**	2.8	**57.9**	3.8	**57.2**	3.3	**55.4**	2.9	**56.2**	3.1	**55.7**	2.5
	UL	**62.4**	2.7	**62.7**	2.5	**62.8**	3.1	**66.2**	3.0	**63.8**	2.5	**64.2**	2.4
SP_s [%]	LL	**37.1**	2.3	**38.1**	2.8	**36.3**	30	**36.3**	2.0	**36.0**	2.7	**35.9**	1.6
	UL	**42.5**	3.3	**43.2**	3.6	**42.9**	3.0	**45.5**	3.0	**43.7**	3.0	**45.5**	1.9
SP_d [%]		19.8	2.9	19.7	4.6	20.4	4.4	18.9	2.5	20.1	3.6	19.4	3.4
SI [%]		10.2	6.7	8.3	6.3	9.7	6.0	15.5	6.6	12.8	7.8	14.2	4.2
WS [m/s]		1.03	0.07	0.98	0.08	1.05	0.10	0.99	0.10	0.97	0.11	1.08	0.12

Mean and standard deviation for the spatiotemporal parameters of loaded (LL) and unloaded (UL) leg for children with CP. During gait without added masses (NG), LL represents the more affected limb of the children with CP. For each variable, the significant differences between LL and UL are reported in bold characters.

### Kinematic Data

**Figure 2:**
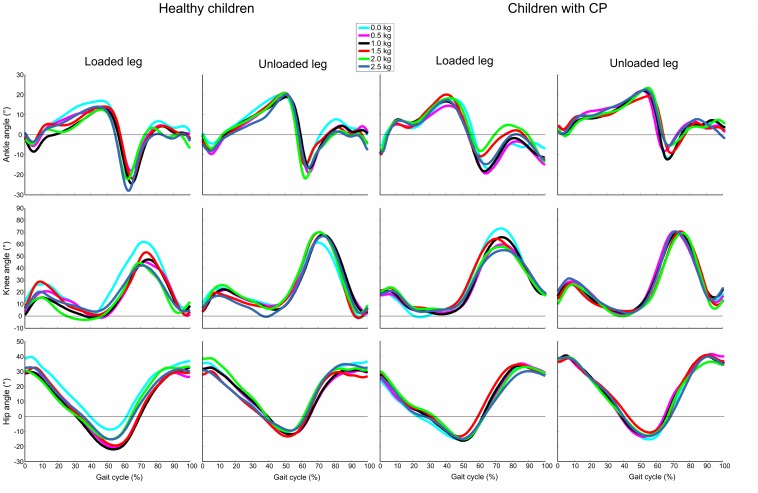
Hip, knee and ankle angle time histories. Time-normalized plot of hip, knee and ankle angles collected from a representative healthy child and a representative child with CP for the six trial conditions (see legend inside the figure). The angle trends are reported for UL and LL.

**Figure 3:**
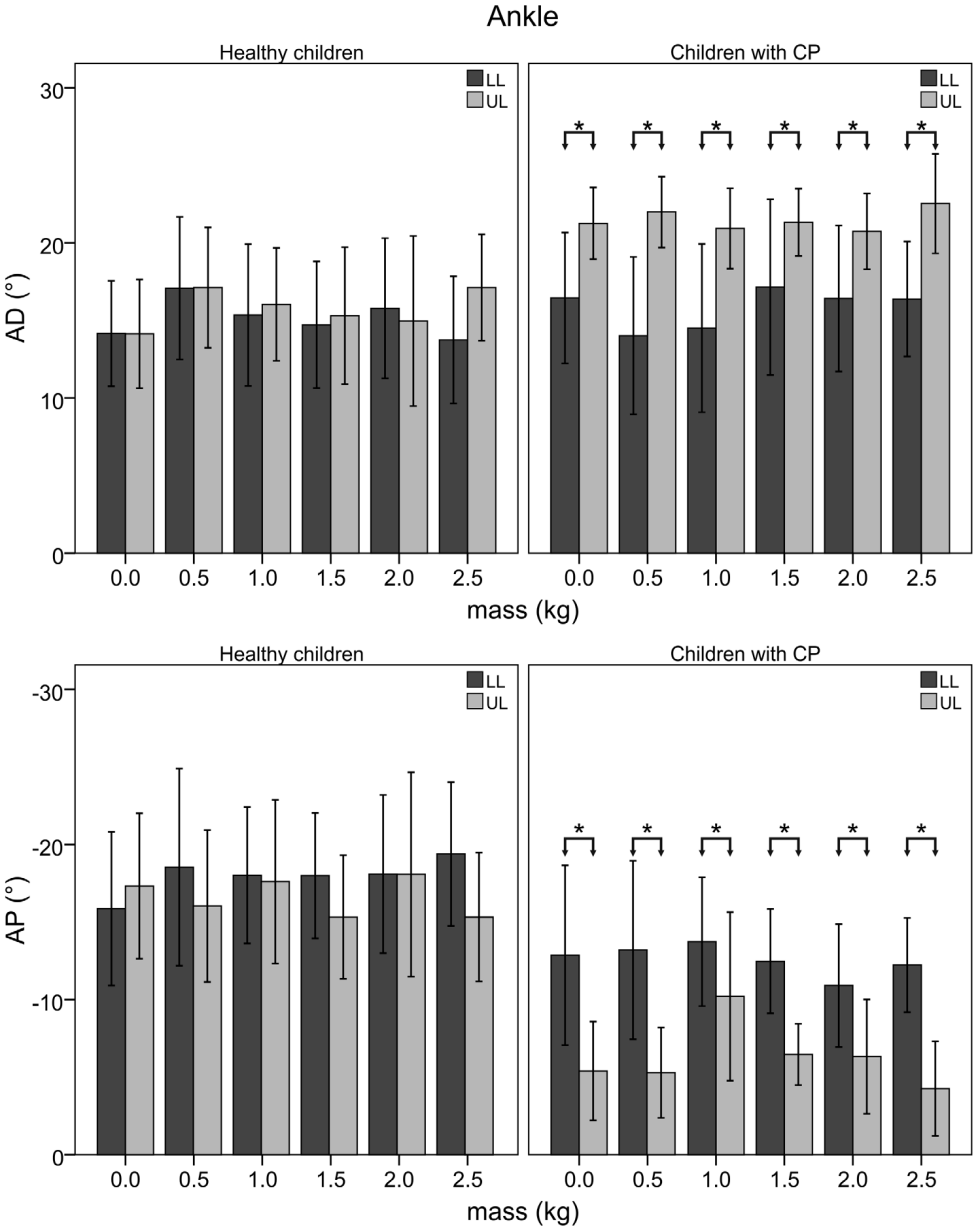
Peaks of ankle angle of loaded and unloaded leg. Mean and standard deviation for peak values of ankle dorsiflexion (AD) and plantarflexion (AP) angles of loaded (LL) and unloaded (UL) leg for healthy children and those with CP as a function of the added mass value. Asterisks indicate significant differences.

**Figure 4:**
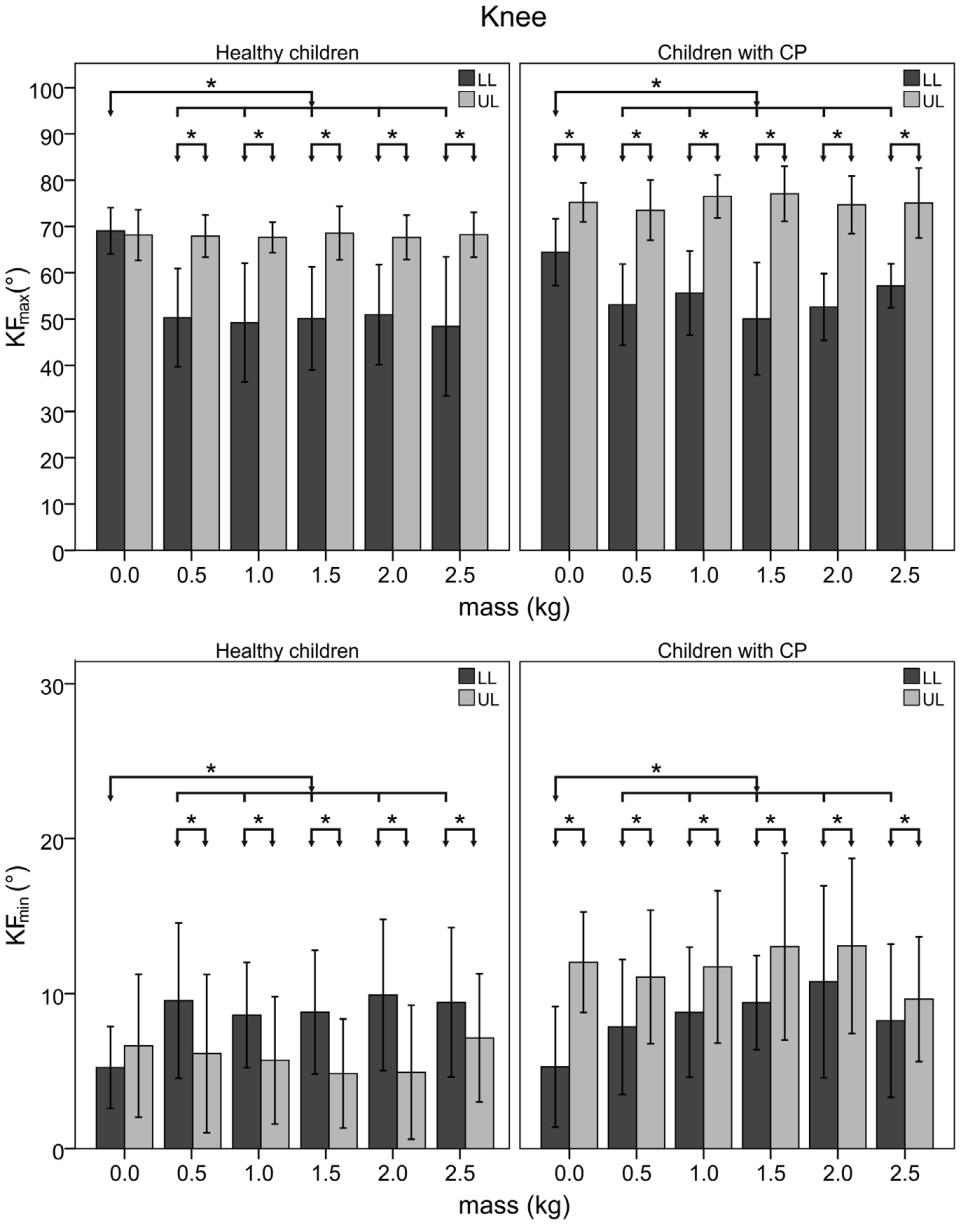
Peaks of knee angle of loaded and unloaded leg. Mean and standard deviation for peak (KF_max_) and lowest (KF_max_) values of knee flexion angle of loaded (LL) and unloaded (UL) leg for healthy children and those with CP as a function of the added mass value. Asterisks indicate significant differences.

**Figure 5:**
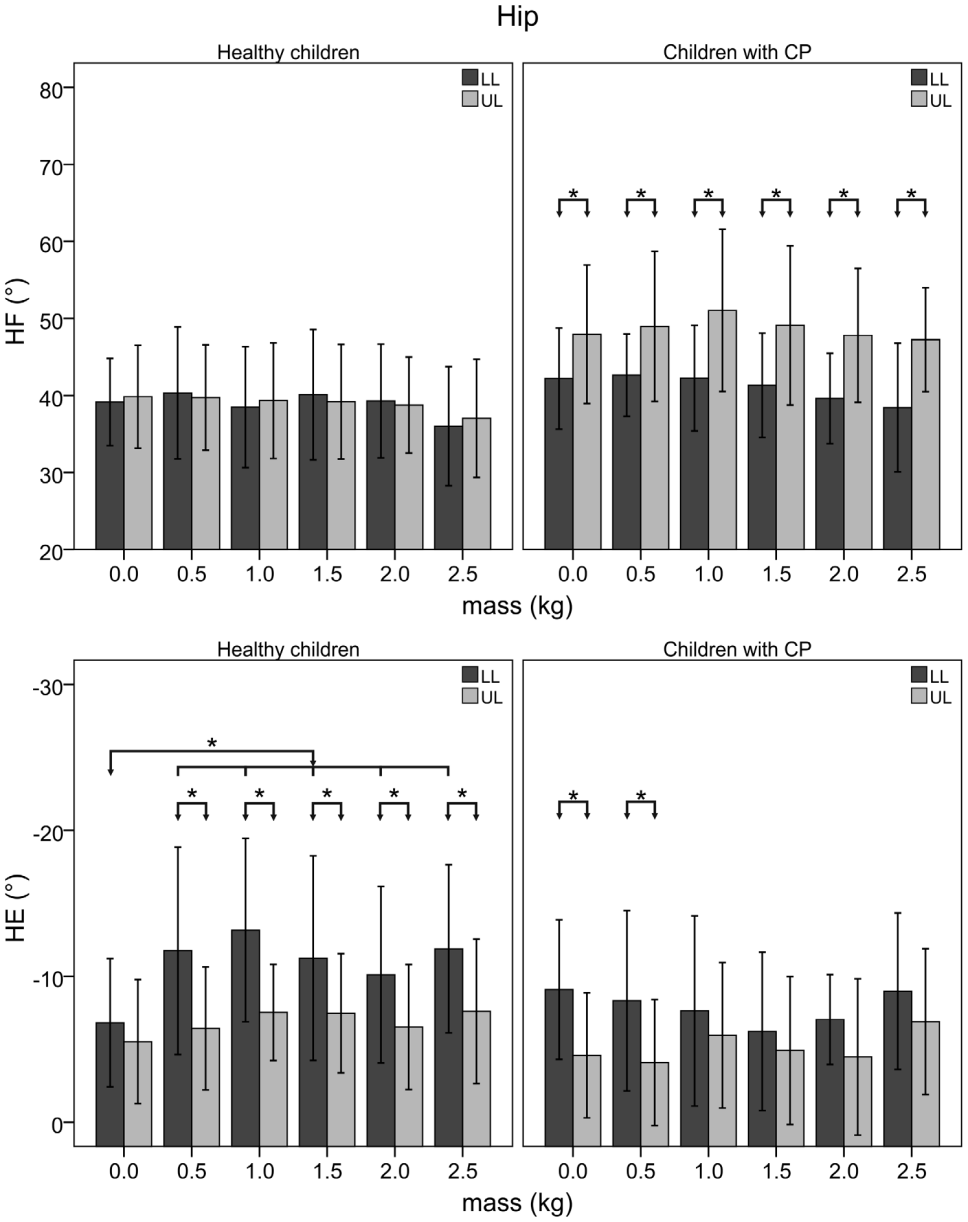
Peaks of hip angle of loaded and unloaded leg. Mean and standard deviation for peak values of hip flexion (HF) and extension (HE) angles of loaded (LL) and unloaded (UL) leg for healthy children and those with CP as a function of the added mass value. Asterisks indicate significant differences.

Figure 2 shows the joint rotations of representative trials collected with a healthy child and a child with CP. Figure 3, Figure 4, and Figure 5 illustrate the statistical analysis of each kinematic parameter for healthy children and children with CP. For the loaded leg of healthy subjects, KF_max_ (p<0.001), KF_min_ (p<0.01) and HE (p<0.001) were significantly different between “natural gait” and the other “with added masses” conditions. In particular, KF_max_ decreased by 30%, KF_min_ increased by a factor of two, and HE increased by 50% with the added masses on the knee. Moreover, the same kinematic parameters were significantly different between LL and UL (p<0.01) except for the NG condition. For the loaded leg of children with CP, a significant difference among trial conditions was found for KF_max_ (p<0.05) and KF_min_ (p<0.05). More specifically, the value of KF_max_ decreased by 20% and KF_min_ increased up to a factor of two between “natural gait” and the other “with added masses” conditions. Statistical differences were found between LL and UL (p<0.05) for each kinematic parameter except for the HE index during 1.0 kg (p = 0.40), 1.5 kg (p = 0.48), 2.0 kg (p = 0.16) and 2.5 kg (p = 0.36) conditions. There were no significant differences among conditions for healthy and CP subjects’ unloaded leg.

## Discussion

### Healthy Children

From the results, it was determined that the spatiotemporal parameters did not change as a function of the mass increase up to 2.5 kg. In particular, the walking speed of normally developed participants did not significantly change. This result replicates Barnett et al. (1993), who found irrelevant differences of walking speed between unweighted and weighted conditions. Our data suggest that adding mass to a healthy child’s knee does not alter either the stance phase or the symmetry index; this result is similar to adult results reported in Noble et al. (2006). There was no significant gait asymmetry for healthy children, as shown by a value of SI close to zero during each trial condition. SP_s was not different among trials both for LL and UL and it entails a high ability of children to support an increase of the weight applied to the swinging leg. Moreover, the presence of the added masses does not alter the step length of loaded and unloaded leg of children.

Examining the knee flexion and hip extension for the LL, we observed that the knee brace (0.5 kg condition) is quite restrictive and has a significant impact on the joint angles as compared to unconstrained gait. Moreover, the angles did not change when adding up to 2.5 kg to the knee brace and, consequently, the mass increase has no additional impact on the gait. Therefore, one can conclude changes in gait were induced by the knee brace mechanical constraint rather than by the associated mass increase.

Healthy subjects had a greater hip extension during the last stance phase, observed as well in adults by Noble et al. (2006). This increase in hip extension can be ascribed to a compensatory mechanism generated in order to absorb the reduction in knee extension due to the knee brace limitation. The effects of knee brace are evident also from the significant differences of knee flexion and hip extension between UL and LL except for the NG condition.

Regarding the ankle joint, the presence of the knee brace and lead masses had no statistical effect on the angle values as also reported by Noble et al. (2006).

Our findings are quite distinct from the results reported by Gordon et al. (2006). The researchers analyzed walking with and without an ankle-foot orthosis (1.6 kg) positioned on the ankle and they did not find significant differences in the lower limb joint kinematics. We speculate that different findings could be attributed to the different mass position and, consequently, to the different mechanical constraint acting on the lower limb kinematics. Our overall analysis leads us to hypothesize that limiting ankle kinematics with an ankle-foot orthosis does not generate any compensatory mechanism in the upper joints of legs, i.e., knee and hip. Instead, our study suggests that children walked with a greater hip extension due to the knee brace constraint. The different behavior may be attributed to the different constrained joint. In fact, the perturbation of the knee, which represents the intermediate joint of the lower limb kinematic chain, entails the need for an adjustment of the other joints, particularly during the stance phase. We speculate that the ankle perturbation does not require any modification of the lower limb kinematic chain since the foot is the last body segment of the chain.

The UL kinematic parameters were not affected by the unilateral loading on the other leg. Moreover, we did not observe any statistical differences among different trial conditions at the hip, knee and ankle.

### Children with CP

We observed no statistically significant differences in the spatiotemporal parameters among the various conditions. These findings are consistent with those of Khanna et al. (2010), who reported similar results in WS and SP for post-stroke adult patients. Children with CP had a significantly greater SI than healthy children for any loading condition, highlighting typical gait asymmetries in this population. These asymmetries were also confirmed by the significant difference on SP and SP_s between LL and UL. Moreover, the ability of children to support an increase of weight of the swinging leg was confirmed also for children with CP because SP_s did not statistically change. Therefore, the swing phase of the more affected leg did not change with its loading increase. The peak of knee flexion angle decreased and the lowest value of the same angle increased with the knee brace and with the increase in mass. Moreover, KF_max_ and KF_min_ did not change when adding masses on the knee brace up to 2.5 kg. We believe this result highlights the influence of the knee brace constraint rather than the increased loading and mirrors the results obtained with healthy children. Conversely, the kinematics of the hip in children with CP did not change when they wore the knee brace, as occurred in healthy subjects. This result leads us to speculate whether children with CP are unable to select a proper hip compensatory mechanism which would absorb the change in knee angle. It could imply a lower ability in balance control during the gait and, consequently, it could explain the higher gait asymmetry highlighted by the slight SI increase as a function of the added masses. Therefore, children with CP exhibit a lower ability in adapting to external perturbations [33].

The kinematics of the ankle joint did not change with the presence of the knee brace and lead masses. This finding is in contrast to results provided by Khanna et al., who reported the decrease in ankle dorsiflexion when the subjects walked wearing a robotic device [26]. The different ankle behavior may not be attributed to the position of the mass because it was placed on the proximal third of leg in both studies. Nevertheless, the robotic device is characterized by two linear actuators connecting the knee brace to the orthopedic shoe [15], [26] and, consequently, the different result might be due to the mechanical constraint acting on the ankle. Moreover, the different age (children vs. adults) and pathology (CP vs. stroke) of the examined populations could be the reason for the difference in ankle angle.

The kinematics of the unloaded leg was not altered by the presence of added masses on the contralateral limb with no variation of joint angles at each trial condition. For almost all the variables, the statistical differences between LL and UL confirmed the typical gait asymmetries in subjects with CP. Nevertheless, we did not find any difference between UL and LL for HE index during the 1.0 kg, 1.5 kg, 2.0 kg and 2.5 kg conditions. This different behavior is due to the decrease of HE index gap between LL and UL generated by non-significant decrease and increase of hip angle peak for LL and UL, respectively.

### Study Limitations

A limitation of the study was that we evaluated the added mass mounted at the knee which might be of limited value with the design of other devices having different loading characteristics. In addition, the system here used, composed by a brace and added masses, is passive while wearable robotic systems synergistically and dynamically interact with the patient to compensate gait deficiencies. Nevertheless, our results might be relevant for designers developing similar technology to assist on gait.

Another limitation inherent in the present study was that we did not evaluate changes in metabolic demand due to the added mass. However, one must be cognizant that we can only properly evaluate such a demand once the design is completed and the assistance of actuators can be accounted for.

## Conclusions

In conclusion, the central finding of this study is that adding a mechanical constraint alters some biomechanical parameters in gait, but adding a mass up to 2.5 kg at the proximal third of leg does not alter the lower limb kinematics. As a result, a wearable robotic device mounted at knee level weighing 25 N or less will not noticeably modify the gait patterns beyond the impact of the knee brace itself.
